# The structural and microbiological properties of human cadaveric iliac vessel grafts stored at a readily available standard freezer: a comprehensive analysis as a function of storage time

**DOI:** 10.3389/fsurg.2026.1752062

**Published:** 2026-03-12

**Authors:** Abdullah Boga, Fuat Aksoy, Ercüment Gürlüler, Halit Ziya Dundar, Fatih Celik, Ozkan Balcin, Ceren Oy, Bilge Arıcan, Zehra Minbay, Feriha Ercan, Ekrem Kaya

**Affiliations:** 1 Department of General Surgery, Bursa Uludag University Faculty of Medicine, Bursa, Türkiye; 2 Department of Pediatric Surgery, Bursa Uludag University Faculty of Medicine, Bursa, Türkiye; 3 Clinics of General Surgery, Bursa City Hospital, Bursa, Türkiye; 4 Department of Histology and Embryology, Bursa Uludag University Faculty of Medicine, Bursa, Türkiye; 5 Department of Histology and Embryology, Marmara University Faculty of Medicine, Istanbul, Türkiye

**Keywords:** organ transplantation, standard freezer, storage time, vascular allografts, iliac vessel

## Abstract

**Background:**

Vascular allografts are very important tool for transplantation procedures especially in living donor liver transplantation (LT). The aim of this study is to evaluate the histopathological and microbiological properties of human cadaveric iliac vessel grafts stored by a readily available method (freezing at −24 °C without using cryoprotectant solution) and to determine the impact of storage time on these parameters.

**Methods:**

Donor characteristics, histopathological changes on light microscopy [tunica intima, internal elastic lamina (IEL), tunica media in artery allografts], scanning electron microscopy (SEM) endothelial morphology grade and the microbiological results were recorded.

**Results:**

A total of 54 cadaveric iliac vessel grafts (28 iliac arteries and 26 iliac veins) harvested from 28 donors were grouped based on the storage period as fresh control (0–24 h, *n* = 10) and 0–6 months (*n* = 10), 6–12 months (*n* = 10), 12–24 months (*n* = 12) and >24 months (*n* = 12) storage groups. Demographic data of the donors were similar along the groups. Some morphological changes were seen in graft stored for >24 months than those stored shorter time on the histopathological examinations and morphometric analysis. Endothelial structure damage was observed less in the grafts those stored shorter than 12 months than longer time in SEM examination. None of the graft samples showed bacterial growth after incubation.

**Conclusions:**

In conclusion, our findings revealed that iliac vessel allografts stored for less than 12 months had the lower risk of morphological, structural and degenerative endothelial changes. Hence, this simple and readily available low cost storage method seems to offer a favorable alternative in allograft storage up to 12 months.

## Introduction

Living donor liver transplantation (LDLT) can be associated with more challenging vascular reconstruction than cadaveric transplantation and a requirement for several vascular grafts (1, 2). Harvested iliac artery and veins from deceased donors are the main source. Use of effective storage methods is critical for continuous source of vascular allografts (VA) for active liver transplantation centers (2–6).

Several techniques for graft storage are available such as cold storage at +4 °C in saline, slow freezing and cryopreservation (2, 4, 7, 8). Amongst these, cryopreservation has become an accepted method and cryopreserved vascular allografts (CVAs) stored at −150 °C in the vapor phase of liquid nitrogen can be kept for 10 years at the tissue bank (6, 9–11). However the allograft storage is a challenging procedure expected to maintain without inducing endothelial injury or immunologic changes to protect the viability or patency of graft and to maintain absolute sterility. Although preservation of the basic collagenous network of the blood vessels, low risk of infection transmission and low immunogenicity prompted the use of CVAs, cryopreservation has been performed with caution due to potential risk of structural changes within the extracellular matrix and early rupture as well as methodological limitations (12–16).

The storage time of CVAs is also suggested to influence resulting in less favorable post-implantation outcome (2, 13). The functional properties and structural damage caused by cryopreservation and prolonged storage of CVAs on histological changes has not been thoroughly investigated and reported mainly for animal models (13, 19, 20). The optimal preservation techniques and storage time for allogeneic VA remains controversial (2, 21, 22).

This study aimed to evaluate the histopathological and microbiological properties of human cadaveric iliac vessel grafts stored by this readily available method (freezing at −24 °C without using cryoprotectant solution) and to determine the impact of storage time on these parameters.

## Materials and methods

The experimental work was performed only on allografts harvested from donor and removed from the tissue bank as “non-used but suitable” (these are all surplus) for transplantation. The study was conducted in accordance with the ethical principles stated in the “Declaration of Helsinki” and approved by the Bursa Uludag University Ethics Committee without the need for inform consent (Date:18/08/2020, Protocol No: B.30.2.ULU.0.20.12.01-40-02.899). An official written Ethics Committee decision letter explicitly confirming the waiver of written informed consent for this study has been obtained from the Bursa Uludağ University Clinical Research Ethics Committee and is provided as Supplementary Material.

Microbiological cultures for aerobes, anaerobes and fungi were performed on cross-sectional tissue specimens prepared from the thawed grafts. The histopathological evaluation included internal elastic lamina (IEL) integrity, tunica media thickness and morphological examination under the light microscope. For the evaluation of endothelial morphology, the samples were examined under scanning electron microscope (SEM) and graded via a scoring system (24).

Aseptic techniques are used during harvesting VAs transported in a sterile container with the organ preservation solution (UW solution) to the transplantation center. Fat tissues around the vessel were removed and vessel lumen was rinsed with cold saline (0.1–10 °C),while grafts were also inspected for patency in the back-table. Fresh grafts not used for transplantation, were subjected to storage procedure. The VAs were rinsed and placed within solution containing 1 g cefazoline sodium (10 mg/mL) in 100 mL saline in a sterile container and were kept in freezer at −24 °C as per storage periods defined in the study protocol.

Each group of grafts were thawed in 0.9% NaCl solution at 37 °C at the end of storage time. After complete loss of ice, the VAs were rinsed in three rounds of saline on sterile operating table. A 5 mm full-layer part of each graft was placed in thioglycolate medium for microbiological evaluation, while tissue samples prepared via 1 cm circumferential and longitudinal histological sections were fixed in 10% formaldehyde solution for 48 h.

For microbiological evaluation, samples in thioglycolate medium were incubated at 36°C and examined after 24 h, 48 h, and beyond 48 h (>48 h) for microbial growth, as demonstrated by the appearance of turbidity. The growth detected mediums were further examined for aerobic, anaerobic and fungus isolates via MALDİ-TOF (Bruker, ABD), API-ID 32C (BioMérieux, France) systems after sub-cultured onto selective and nonselective plated media or appropriate anaerobic plated media.

For histological analysis, thawed vessel specimens fixed in 10% formaldehyde solution for 48 h were trimmed and processed for routine histopathological examination and paraffin blocks were sectioned at 5 µm thickness. On light microscopy (Olympus BX50), serial sections were evaluated for general morphology after Hematoxylin—Eosin (H&E) staining and for internal elastic lamina and connective tissue structures after Verhoeff's staining and scored (1 to 4) via IEL scoring system (Table 1). Tunica media thickness was measured via the Verhoeff method in stained transverse section using 20× objective and lab Sens (version 1.1; Olympus Soft Imaging Solutions, Hamburg, Germany) software program. Measurements were taken between the internal elastic lamina and surface of external elastic lamina adjacent to tunica media in arteries, and between the subendothelium-tunica media border and tunica media-adventitia border in veins. The arithmetic means of 120 thickness values obtained for each sample was considered the final tunica media thickness (24). For the evaluation of endothelial morphology, 3 × 4 mm thawed vessel samples primarily fixed in 10% formaldehyde solution were further examined at least 10 similar fields under scanning electron microscope (SEM, JEOL JSM-5200) and endothelial integrity and superficial morphology of endothelial cells were graded (1 to 6) using SEM endothelial morphology scoring system (Table 1) (18).

**Table 1:** Scoring systems used for assessing internal elastic lamina structural changes and superficial morphology of endothelial cells (24).

IEL score	IEL structural changes (fragmentation, reduplication)
1	Morphologically intact IEL
2 (mild)	Structural changes involving ¼ of vessel diameter
3 (moderate)	Structural changes involving ¼–½ of vessel diameter
4 (severe)	Structural changes involving more than ½ of vessel diameter
SEM score	Superficial morphology of endothelial cells
Grade 1	Morphologically intact endothelium—putative physiological changes are not reflected in the superficial morphology of endothelial cells
Grade 2	Confluent endothelium with structural inhomogeneity—irregularities in the form of individual cells and changes of their membranes are detectable
Grade 3	Disruption of intercellular contacts—continuity of endothelial coverage is lost, and endotheliocytes shrink while still adhering to basal membrane
Grade 4	Separation of endothelial cells—endotheliocytes separated from the basal lamina. Initially, they protrude by their intercellular edges into the lumen
Grade 5	Complete loss of endothelium—denudation of the endothelial covering with the basal lamina exposed
Grade 6	Damage of subendothelial layers—the luminal surface is covered only by remnants of basal membrane, and the fiber structure of the lamina fibrosa and the lamina ventricularis may be dissolved

IEL, internal elastic lamina; SEM, scanning electron microscopy.

### Replicates and measurements

All analyses were performed using independent biological samples, with each vascular graft considered as one experimental unit. For histopathological evaluation, serial sections were obtained from each graft, and quantitative measurements were averaged per graft for statistical analysis. For scanning electron microscopy, at least 10 independent microscopic fields per graft were evaluated for endothelial morphology grading. Microbiological analysis was performed using one representative full-layer tissue sample from each graft.

### Statistical analysis

Statistical analysis was made using IBM SPSS Statistics for Windows, version 26.0 (IBM Corp., Armonk, NY). Fisher's exact test was used for the comparison of categorical data. Kruskal–Wallis *H*-test was used for the parametric variables. Data were expressed as median(minimum-maximum) and percent (%) where appropriate. *p* < 0.05 was considered statistically significant.

## Results

### Study samples and analysis overview

A total of 54 cadaveric iliac vessel grafts (28 iliac arteries and 26 iliac veins) harvested from 28 donors (age:20 to 89 years) declared brain dead, determined as suitable for organ transplantation and underwent cadaveric organ donation were included in this study (between January 2018 and June 2021). These iliac vessel allografts were grouped based on the storage period, as fresh control (0–24 h, *n* = 10,5 arteries and 5 veins) and 0–6 months (*n* = 10,5 arteries and 5 veins),6–12 months (*n* = 10,6 arteries and 4 veins), 12–24 months (*n* = 12, 6 arteries and 6 veins) and >24 months (*n* = 12,6 arteries and 6 veins) storage groups.

Demographic characteristics of donors were similar across study groups, while active smoking was significantly more common in VAs stored for 6–12 months compared to those stored for 0–6 months and control (*p* = 0.002 and *p* = 0.015,respectively) (Table 2).

**Table 2:** Donor characteristics in iliac vessel allograft groups.

Donor characteristics	Fresh	Storage duration 0–6 months	Storage time 6–12 months	12–24 months	(>24 months)	*P* value
	(*n* = 5)	(*n* = 5)	(*n* = 6)	(*n* = 6)	(*n* = 6)	
Age, median (min-max)	65 (20–81)	70 (60–81)	52 (26–57)	58 (42–89)	56 (36–85)	0.196^a^
Gender, *n* (%)						
Female	4 (80.0)	3 (60.0)	2 (33.3)	3 (50.0)	3 (50.0)	0.699^b^
Male	1 (20.0)	2 (40.0)	4 (66.7)	3 (50.0)	3 (50.0)	
BMI (kg/m^2^),						
median (min-max)	30 (22–41)	27 (23–31)	25 (24–27)	25 (21–29)	27 (24–31)	0.601^a^
Active smoking, *n* (%)	0 (0)*	1 (20)*	6 (100)	2 (33.3)	3 (50)	**0** **.** **007** ^b^
Alcohol consumption, *n* (%)	0 (0)	0 (0)	3 (50)	1 (16.7)	0 (0)	0.095^b^
Comorbidities, *n* (%)						
Diabetes	2 (40)	2 (40)	1 (16.7)	1 (16.7)	0 (0)	0.185^b^
Hypertension	3 (60)	4 (80)	4 (66.7)	2 (33.3)	3 (50)	0.675^b^
CAD	1 (20)	1 (20)	0 (0)	1 (16.7)	0 (0)	0.604^b^
COPD	0 (0)	0 (0)	0 (0)	1(16.7)	0 (0)	>0.99^b^

CAD, Coronary artery disease; COPD, Chronic obstructive pulmonary disease.

Bold values indicate statistically significant differences (*p* < 0.05).

^a^ Kruskal–Wallis *H*-test.

^b^ Fisher's exact test.

* *p* < 0.05 compared to 6–12 months’ group.

Histopathological analysis on light microscopy revealed intimal thickening, and structural changes in IEL including fragmentation and duplication in most of iliac artery grafts,as well as degenerative changes in tunica media and sclerosis. But these changes are not significantly different along the groups. Intimal findings ranged from well-protected luminal surface in some grafts to completely endothelium-deficient areas in others. On morphometric analysis of artery grafts for IEL, no significant difference was noted between groups. Score 4 reduplication was more common in the grafts stored for >24 months (83.3%) compared to those stored less than 24 months (0.0%) (*p* < 0.05). No significant difference was noted in tunica media thickness in artery grafts (Table 3). Tunica media thickness of arterial grafts showed no statistically significant difference among storage groups (median [min–max]: fresh 361 [286–639] µm, 0–6 months 296 [242–546] µm, 6–12 months 311 [290–528] µm, 12–24 months 412 [234–439] µm, and >24 months 233 [188–324] µm; *p* = 0.075).

**Table 3:** Morphometric analysis: IEL scoring and tunica media thickness.

IEL scoring, *n* (%)	Fresh	Storage duration 0–6 months	Storage time 6–12 months	12–24 months	(>24 months)	*P* value
	(*n* = 5)	(*n* = 5)	(*n* = 6)	(*n* = 6)	(*n* = 6)	
Fragmentation						
Score 1	0 (0)	1 (20)	0 (0)	1 (16.7)	0 (0)	0.830^a^
Score 2	4 (80)	4 (80)	5 (83.3)	4 (66.7)	6 (100)	
Score 3	1 (20)	0 (0)	1 (16.7)	1 (16.7)	0 (0)	
Score 4	0 (0)	0 (0)	0 (0)	0 (0)	0 (0)	
Reduplication						
Score 1	0 (0)	2 (40)	1 (16.7)	0 (0)	0 (0)	**0**.**006**^a^
Score 2	3 (60)	0 (0)	3 (50.)	4 (66.7)	0 (0)	
Score 3	2 (40)	2 (40)	2 (33.3)	2 (33.3)	1 (16.7)*	
Score 4	0 (0)	1 (20)	0 (0)	0 (0)	5 (83.3)*	
Tunica media thickness (µm), median(min-max)						
Artery grafts	361 (286–639)	296 (242–546)	311 (290–528)	412 (234–439)	233 (188–324)	0.075^b^
Vein grafts	60 (38–99)	53 (38–87)	78 (37–98)	45 (35–99)	80 (37–112)	0.666^b^

IEL, internal elastic lamina (for arterial grafts).

Bold values indicate statistically significant differences (*p* < 0.05).

^a^ Fisher's Exact test.

^b^ Kruskal–Wallis *H*-test.

* *p* < 0.05 compared to other groups (excluding 0–6 months). Reduplication scores was more commonly seen in the graft stored >24 months’ group then the others.

**Figure 1:**
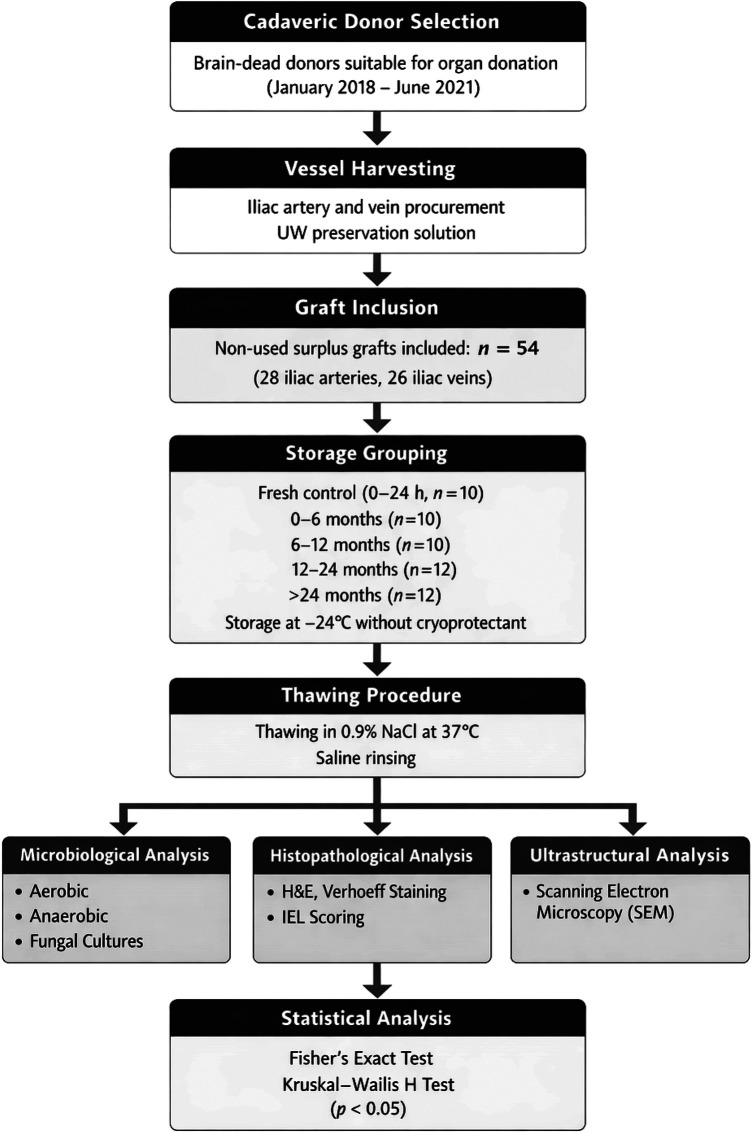
Flowchart summarizing the experimental workflow of the study, including cadaveric donor selection, iliac vessel graft procurement, storage grouping according to preservation duration, thawing procedure, microbiological, histopathological and ultrastructural analyses, and statistical evaluation.

**Figure 2:**
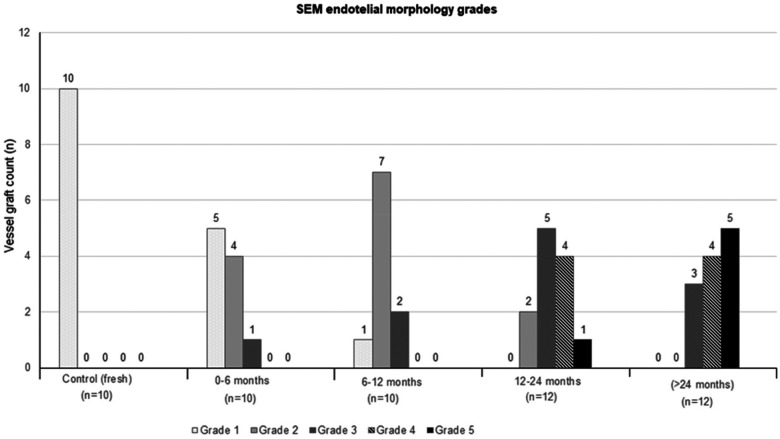
Morphological scores of the grafts (both arterial and vein) in SEM examination. All of the control (fresh group) grafts have intact epithelium. Grade 1 morphology was significantly higher in the fresh grafts (100.0%) compared to the other groups (50.0%,10.0%,0.0% and 0.0%, in 0–6 months,6–12 months,12–24 months and >24 months’ groups respectively, *p* = 0.003 in 0–6 months and *p* < 0.001 for other groups). Endothelial damage is beginning from group 2 (0–6 months) and increase along to the groups, and it is clearly observed in group 4,5 (12–24 month, >24 month). Significant endothelial damage (grade 4 and 5) is seen in groups stored longer than 12 months (*p* < 0,02).

**Figure 3:**
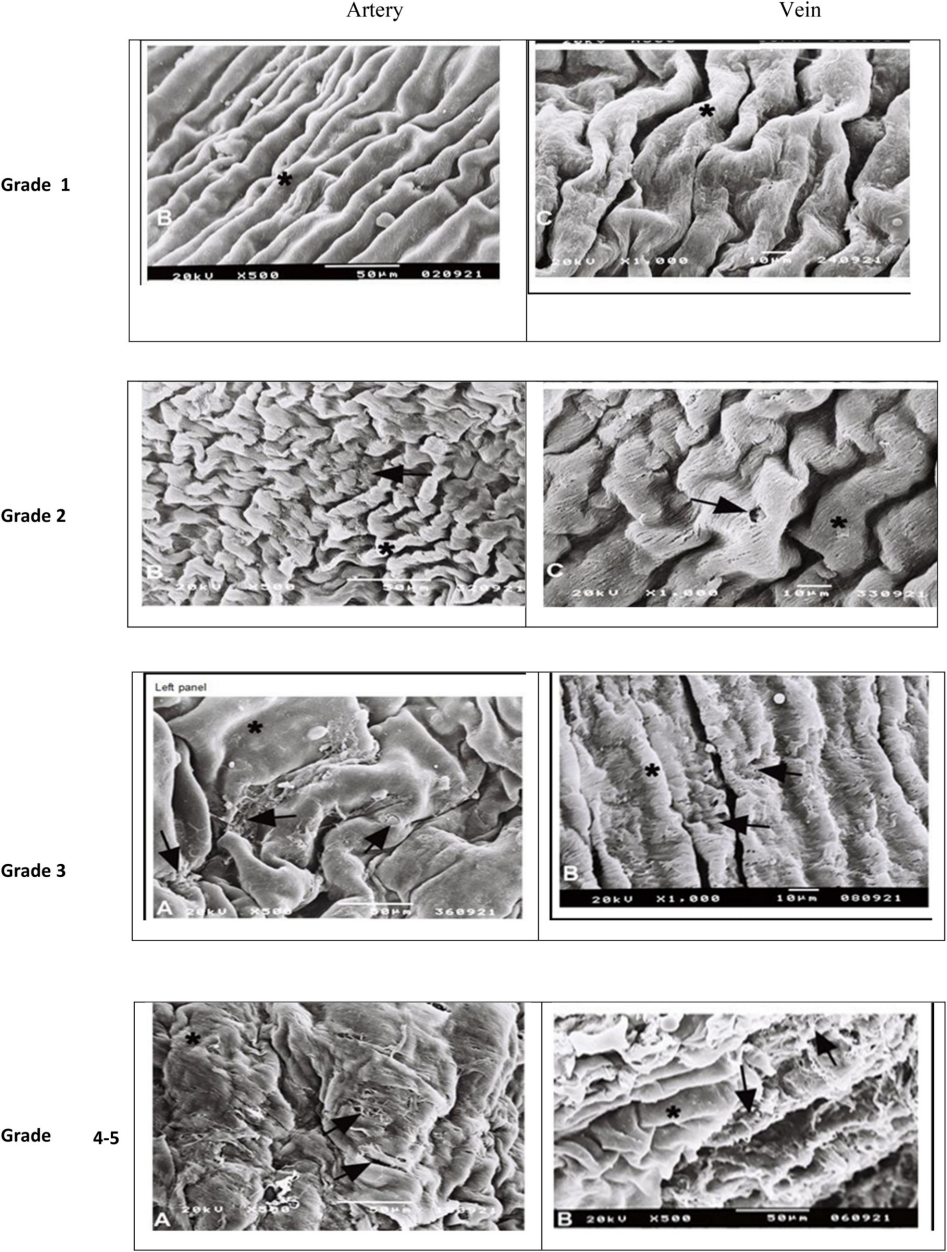
Endothelial layer morphology in SEM. Arterial pictures are in left side and vein pictures are in right side. *: natural morphology. **‣:** Disruption of intercellular morphology and irregular morphology (grade 2), loss of endothelial continuity and separation of epithelium in patches (grade 3), appearance of endothelial layer in patches (grade 4), complete loss of epithelium disseminated appearance of subendothelial layer (grade 5).

All graft samples in the control group had morphologically intact endothelium. The other stored groups’ SEM grade are shown in Figure 2. Grade 1 morphology was significantly higher in the fresh grafts compared to the other groups (*p* = 0.003 in 0–6 months and *p* < 0.001 for other groups). Various degree endothelial damages were seen in along the groups 2–5 and these morphological changes is increasing in the beginning from the 0–6 months’ group to >24 months’ group. It is clearly observed in groups 4 and 5. endothelial damage were statistically worse in the groups stored longer 12 months than shorter (*p* < 0,02, Figures 1–3).

None of the graft samples incubated at 36°C showed turbidity indicating microbial growth after 24 h, 48 h, or beyond 48 h (>48 h).

## Discussion

Long term patency of the vascular grafts’(especially arterial grafts) anastomosis is directly related to graft and patient survival in the transplantation field. A prototypical arterial allograft is expected to maintain anatomical and physiological structure of a native artery including arterial wall viscoelastic and inertial properties (18). Although the impact of cryopreservation and subsequent thawing on these properties remain controversial (18, 25), fresh freezing of the graft to −80 °C is considered likely to cause structural changes within the extracellular matrix which may adversely affect the quality of the allograft and long-term outcomes after its utilization (13). Cryopreservation of arteries was also associated with impaired reactivity, endothelial integrity and function as well as damages on the smooth muscle cells, elastin, and collagen fibers and strain and stress in the arterial wall (18, 25–29).

In this study, iliac vessel allografts stored at a readily available laboratory freezer at −24 °C without use of a cryoprotectant solution were assessed in terms of potential histopathologic, mechanic and microbiologic alterations as a function of storage time. SEM endothelial morphology analysis revealed grade 2 to grade 5 changes in stored iliac vessel grafts compared to fresh grafts, which were also in favor of grade 2 morphology in grafts stored for less than 12 months but were consistent with grade 3 to 5 morphology in those stored for more than 12 months (23). Changes in tunica intima (intimal thickening, atheroma plaque) and structural changes in IEL (fragmentation, reduplication) and edema in tunica media were evident even in fresh grafts and were similar across storage groups. Degenerative changes in tunica media (hyalinization, of connective tissue and sclerosis) were not seen in fresh grafts and were more prominent in grafts stored for more than 6 months. In an animal experiment study involving arterial grafts showed a complete loss of the muscle component of the tunica media along with the formation of a stable neointima in CVAs in long-term implants (31). In a human CVA study (iliac veins and iliac arteries),while no significant change was noted between those stored for ≤1 year and for >1 year in terms of structural changes or the patency of CVAs in LDLT,38% of arterial CVAs had different degrees of intimal thickening and atherosclerotic change (17). In another study assessing the effect of cryopreservation on the structure of vessel wall as a function of storage time in porcine aortic grafts, although histological examination revealed no significant changes in the structure of aortic wall at different time points of storage, a short storage time is suggested given the minor alterations in microstructure of fibers noted in the first three months (13).

Our findings indicate that iliac vessels grafts stored for less than 12 months seem to be more advantageous than those stored for longer periods in terms of lower risk of advanced (grade 3 to 5) changes in SEM endothelial morphology, and lower rates of fragmentation and duplication in IEL and medial sclerosis. Hence, given the higher risk of impaired endothelial structure with storage times prolonged over 12 months and the finding that grafts stored for less than 12 months seem to have the closest morphological appearance to fresh grafts, our findings seem to indicate favorable utility of freezing storage of iliac vessel allografts in a readily available laboratory freezer at −24 °C without use of a cryoprotectant solution. Likewise, in a study at a high-volume liver transplant center, storage of 29 allogeneic vascular graft (16 saphenous vein, 10 iliac vein, and 3 iliac artery grafts) at −22 °C for a maximum of 3 months without any preservation solutions or antimicrobials was reported to yield successful vascular reconstructions and no bacterial growth in any tissue samples (2). Although this study was not a comparative study in terms of cost analyses, it is clear that this technique cheaper than cryopreservation.

Endothelial continuity or protection of endothelium is considered one of the most important factors in storage of vascular allografts, in terms of potential damage to vascular structures enabling viscoelastic properties of vessel and consequent development of aneurysm and rupture, as well as induction of early thrombosis which is one of the most feared complication (18, 19, 30–35). In an experimental porcine model with aorta and vena cava allografts stored in 75% ethanol solution for 12 months at 4 °C, histopathological analysis revealed normal elastic pattern but degeneration of endothelial cells after preservation. Nonetheless, after implantation, he formation of the endothelium cell-like layer was seen in both aorta and vena cava allografts, indicating that vascular allografts were functional (36). In our study, endothelial damage was significantly worse after 12 months’ preservation, but fresh stored arterial grafts’ endothelium was intact in SEM examination. Therefore, we can say that storage time is an important factor for the development of endothelial injury.

In the present study, we used the same thawing method in all groups and cannot make any comment of thawing. Various thawing procedures were tested before and there were no important differences between each other in terms of SEM morphology, endothelial integrity and biomechanical properties of the vessels in the previous studies (18, 37).

Protection of dynamic properties of vessel depends on viable smooth muscle and integrity of vascular wall elastic lamella and collagen. Notably, autografts, particularly in patients with atherosclerosis, are considered to fail due to intimal hyperplasia as a result of migration of vascular SMCs from the vessel media to the intima, SMC proliferation and extracellular matrix deposition (38). In the present study, intimal thickening, fragmentation and reduplication in IEL and edema in tunica media were evident in most of the iliac artery grafts regardless of the storage time and even in the fresh grafts. Although tunica media thickness was similar across storage periods in both iliac artery and vein allografts used in our study, iliac artery grafts stored for >24 months showed a tendency for a decreased tunica media thickness. Degenerative changes in tunica media comprised the edema in fresh grafts and all stored graft groups, while hyalinization, connective tissue formation and patch necrotic areas were evident in all artery grafts, particularly in those stored for more than 12 months. Nonetheless, besides the freezing protocol, pre-freezing history of the graft and the timespan between the graft harvest and freezing are also considered responsible for such events including an enhanced probability of vessel wall injury caused by crystal formation during freezing (3, 6, 39).

Our grafts were stored in the presence of 10 mg/mL cefazoline sodium decontamination, and none of the thawed grafts showed signs of aerobic or anaerobic contamination. Nonetheless, the risk of viral transmission is another potential challenge for vascular grafts, and grafts harvested from seropositive and seronegative donors should be stored in separate storage containers (2, 40).

Donor characteristics were similar except for significantly higher rate of active smoking in the group stored for 6–12 months. This may also be related to more extensive changes in tunica intima (atheroma plaque in particular) and tunica media (sclerosis in particular) specifically in this group stored for 6–12 months. Indeed, European Homograft Bank accepts only under 60 year donors without cardiovascular risk factors to eliminate the potential degenerative risks (8). However, in our region, given the limited number of donors and increase in the rates of marginal donor use, vessel grafts without macroscopically disseminated degeneration are also stored. Besides, while the presence of severe atherosclerosis is exclusion criteria for vessel procurement, it is not a contraindication for liver procurement (41).

In conclusion, our findings indicate no risk of bacterial contamination but varying degrees of changes in endothelial histopathology in iliac vessel allografts stored in a freezer at −24 °C without use of a cryoprotectant solution. Allografts stored for less than 12 months had the lower risk of morphological, structural and degenerative endothelial changes and the closest similarity to fresh allograft than those stored for longer periods. Hence, this simple and readily available low cost storage method seems to offer a favorable alternative in allograft storage up to 12 months. In our clinical practice, we prefer vascular graft stored at a standard freezer less than 12 months when we need to use.

### Study limitations

This study has several limitations. First, the relatively small sample size may have resulted in limited statistical power, which may restrict the generalizability of the findings. Second, all vascular allografts included in the analysis were surplus grafts deemed unsuitable for transplantation, and therefore may not fully represent grafts used in routine clinical practice. In addition, thrombosis after vascular anastomosis could not be evaluated, as this study was not designed as an *in vivo* investigation; thus, future *in vivo* studies using grafts obtained from the same donors are warranted. Another limitation is the inability to perform donor matching based on smoking status due to the retrospective nature of the study and limited graft availability. Finally, microbiological assessment was limited to aerobic, anaerobic, and fungal cultures, and viral screening was not performed; therefore, the absence of microbial growth should be interpreted with caution and does not exclude the potential risk of viral transmission.

### Clinical implications

Although clinical outcomes such as long-term graft patency or function were not directly assessed in this study, the observed endothelial and structural alterations may have potential clinical relevance. Endothelial damage and disruption of endothelial continuity are known to contribute to thrombogenicity, impaired vascular reactivity, and early graft dysfunction, which may negatively affect long-term graft patency. Similarly, structural changes such as fragmentation and reduplication of the internal elastic lamina and degenerative changes within the tunica media may compromise the mechanical integrity of the vessel wall, potentially increasing the risk of graft failure or aneurysmal degeneration over time. Therefore, the progressive endothelial and structural deterioration observed with prolonged storage duration may have important implications for graft performance following transplantation and should be considered when selecting vascular allografts for clinical use.

## Data Availability

The original contributions presented in the study are included in the article/Supplementary Material, further inquiries can be directed to the corresponding author.
