# Supplementary material for: The structural and microbiological properties of human cadaveric iliac vessel grafts stored at a readily available standard freezer: a comprehensive analysis as a function of storage time
Source: Front Surg. 2026 Mar 12;13:1752062. doi: 10.3389/fsurg.2026.1752062 (PMC13017798; doi:10.3389/fsurg.2026.1752062)
Supplement: Supplementary file 1 [file Table1.docx]

- - AB, EK > Participated in research design
  - AB, EG, FA, FÇ, EK > Participated in the writing of the paper
  - ZM, FE , EK> Participated in the performance of the research
  - FA, ÖB,EK > Contributed new reagents or analytic tools
  - EG, ÖB, EK > Participated in data analysis

# This paper was presented orally at the 14th Congress of Turkish Transplantation Centers Coordination Association which was held on November 13-15, 2022 in The Turkish Republic of Northern Cyprus.

**Conflict of interest**: The authors declare that they have no conflict of interest.

**Funding:** None
