# Supplementary material for: The structural and microbiological properties of human cadaveric iliac vessel grafts stored at a readily available standard freezer: a comprehensive analysis as a function of storage time
Source: Front Surg. 2026 Mar 12;13:1752062. doi: 10.3389/fsurg.2026.1752062 (PMC13017798; doi:10.3389/fsurg.2026.1752062)
Supplement: Supplementary file 2 [file Table2.docx]

ABP, anastomotic bursting pressure

BMI, body mass index

CVAs, cryopreserved vascular allografts

IEL, internal elastic lamina

LDLT, living donor liver transplantation

LT, liver transplantation

SEM, scanning electron microscope

SMC, smooth muscle cell
