# Supplementary material for: The structural and microbiological properties of human cadaveric iliac vessel grafts stored at a readily available standard freezer: a comprehensive analysis as a function of storage time
Source: Front Surg. 2026 Mar 12;13:1752062. doi: 10.3389/fsurg.2026.1752062 (PMC13017798; doi:10.3389/fsurg.2026.1752062)
Supplement: Supplementary file 3 [file Table3.docx]

**Background**: Vascular allografts are very important tool for transplantation procedures especially in living donor liver transplantation (LT). The aim of this study is to evaluate the histopathological, mechanical and microbiological properties of human cadaveric iliac vessel grafts stored by a readily available method (freezing at -24°C without using cryoprotectant solution) and to determine the impact of storage time on these parameters.

**Methods:** A total of 54 cadaveric iliac vessel grafts (28 iliac arteries and 26 iliac veins) harvested from 28 donors were grouped based on the storage period as fresh control (0- 24 hours, n=10) and 0-6 months (n=10), 6-12 months (n=10), 12-24 months (n=12) and >24 months (n=12) storage groups. Donor characteristics, histopathological changes on light microscopy (tunica intima, internal elastic lamina [IEL], tunica media in artery allografts), scanning electron microscopy (SEM) endothelial morphology grade, anastomotic bursting pressure (ABP), anastomosis diameter and the microbiological results were recorded.

**Results:** Demographic data of the donors were similar along the groups. Some morphological changes were seen in graft stored for >24 months than those stored shorter time on the histopathological examinations and morphometric analysis. Endothelial structure damage was observed less in the grafts those stored shorter than 12 months than longer time in SEM examination. There were no significant differences between ABP of the groups based on the storage time. None of the graft samples showed bacterial growth after incubation.

**Conclusions:** In conclusion, our findings revealed that iliac vessel allografts stored for less than 12 months had the lower risk of morphological, structural and degenerative endothelial changes and the closest similarity to fresh allograft than those stored for longer periods.

Hence, this simple and readily available low cost storage method seems to offer a favorable alternative in allograft storage up to 12 months.

**Keywords:** *Iliac artery allografts; iliac vein allografts; endothelial morphology; standard freezer; storage time*
