# Supplementary material for: The structural and microbiological properties of human cadaveric iliac vessel grafts stored at a readily available standard freezer: a comprehensive analysis as a function of storage time
Source: Front Surg. 2026 Mar 12;13:1752062. doi: 10.3389/fsurg.2026.1752062 (PMC13017798; doi:10.3389/fsurg.2026.1752062)
Supplement: Supplementary file 4 [file Table4.docx]

# The mechanical, structural and microbiological properties of human cadaveric iliac vessel grafts stored at a readily available standard freezer: A comprehensive analysis as a function of storage time

# Abdullah Boga, MD,^1^ Fuat Aksoy, MD, MSc, ^1^ Ercument Gurluler, MD,^1^ Halit Ziya Dundar, MD,^1^ Fatih Celik,MD,^2^ Ozkan Balcin, MD, MSc^3^, Zehra Minbay, MD, PhD, ^4^ Feriha Ercan,MD, PhD ^5^ Ekrem Kaya, MD, ^1^

# 1 Dept. of General Surgery, Bursa Uludag University Faculty of Medicine, Bursa /Turkiye

# 2 Dept. of Pediatric Surgery, Uludag University Faculty of Medicine, Bursa /Turkiye

# 3 Clinics of General Surgery, Bursa City Hospital, Bursa /Turkiye

# 4 Dept. of Histology and Embryology, Bursa Uludag University Faculty of Medicine, Bursa/Turkiye

# 5 Dept. of Histology and Embryology, Marmara University Faculty of Medicine, Istanbul/Turkiye.

# E-mail addresses; [abdullahboga@windowslive.com](mailto:abdullahboga@windowslive.com), [gurluler@gmail.com](mailto:gurluler@gmail.com), [dundarhalitziya@gmail.com](mailto:dundarhalitziya@gmail.com), drfuataksoy@gmail.com, drfatihcelikk@gmail.com, dr.balcin@hotmail.com, zminbay@uludag.edu.tr, eferiha@hotmail.com, ekremkaya@uludag.edu.tr

# Corresponding author: Prof. Ekrem Kaya, M.D.

# Department of General Surgery, Bursa Uludag University Faculty of Medicine, Görükle, 16059 Nilüfer, Bursa, Turkey

# Phone: +90 532 427 05 86

# E-mail: ekremkaya@uludag.edu.tr
