# Supplementary material for: The structural and microbiological properties of human cadaveric iliac vessel grafts stored at a readily available standard freezer: a comprehensive analysis as a function of storage time
Source: Front Surg. 2026 Mar 12;13:1752062. doi: 10.3389/fsurg.2026.1752062 (PMC13017798; doi:10.3389/fsurg.2026.1752062)
Supplement: Supplementary file 5 [file Datasheet1.pdf]

REPUBLIC OF TÜRKİYE
BURSA ULUDAĞ UNIVERSITY
Ethical Committee on Clinical Researches

Ref. No: 57080008-605-260431
Date: 30.01.2026

Subject: Ethical Committee on Clinical Researches Decision dated 02 September 2020 and numbered 2020-15/24

To:
Prof. Dr. Ekrem KAYA
Bursa Uludağ University
Faculty of Medicine
Department of General Surgery
Faculty Member

Reference: Your petition dated 23 December 2025.

Your application submitted to Bursa Uludağ University Ethical Committee on Clinical Researches regarding the study entitled **"Mechanical, Structural Integrity and Microbiological Evaluation of Cryopreserved Human Cadaveric Iliac Vessel Grafts,"** for which you are the principal investigator, was included in the agenda of the meeting dated 13 January 2026 and numbered 2026-1/30 and has been reviewed.

According to the current legislation in our country, all scientific research conducted on humans must commence only after obtaining informed consent that complies with legal regulations and ethical principles, including the Declaration of Helsinki. In non-clinical studies conducted on archival materials such as human biological waste or pathological specimens, it is preferable that the research begin after obtaining informed consent, provided that the patient or their relatives can be contacted. In both cases, the study must be carefully evaluated in terms of ethical principles and an Ethics Committee decision must be obtained. In situations where the patient or their relatives cannot be reached and/or in studies conducted on anonymized specimens/materials, the research may be initiated following Ethics Committee evaluation and approval without the requirement to obtain informed consent.

In the present study, since the cadaveric materials are anonymized and it has been declared that the relatives of the donors cannot be reached, there was no need to obtain informed consent. Our Ethics Committee has evaluated the study from an ethical perspective and has concluded that there is no ethical objection to conducting the aforementioned research.

Submitted for your information and necessary action.

Prof. Dr. Mustafa K. HACIMUSTAFAOĞLU
Chair of Ethical Committee on Clinical Researches

Attachment: Petition of Prof. Dr. Ekrem KAYA

Document Verification Code: kv_InFiqzUma5eiT3y8RUg
Document Verification Address: https://udos.uludag.edu.tr/Teyit/
